# Next-Generation Therapies in Mantle Cell Lymphoma (MCL): The Evolving Landscape in Treatment of Relapse/Refractory After CAR-T Cells

**DOI:** 10.3390/cancers17132239

**Published:** 2025-07-03

**Authors:** Elia Boccellato, Lorenzo Comba, Rita Tavarozzi, Claudia Castellino, Myriam Foglietta, Daniele Mattei, Marco Ladetto, Massimo Massaia, Alessia Castellino

**Affiliations:** 1 Division of Hematology, Santa Croce e Carle Hospital, via Michele Coppino 26, 12100 Cuneo, Italy; boccellato.e@ospedale.cuneo.it (E.B.); comba.l@ospedale.cuneo.it (L.C.); castellino.c@ospedale.cuneo.it (C.C.); foglietta.m@ospedale.cuneo.it (M.F.); mattei.d@ospedale.cuneo.it (D.M.); massimo.massaia@unito.it (M.M.); 2 Transfusion Medicine and Blood Establishment, Santa Croce e Carle Hospital, via Michele Coppino 26, 12100 Cuneo, Italy; 3 Division of Hematology, Santi Antonio e Biagio e Cesare Arrigo Hospital, Spalto Marengo 43, 15121 Alessandria, Italy; rita.tavarozzi@esterni.ospedale.al.it (R.T.); marco.ladetto@ospedale.al.it (M.L.); 4 Department of Translational Medicine, University of Eastern Piedmont, via Paolo Solaroli 17, 28100 Novara, Italy; 5 Laboratory of Blood Tumor Immunology, Molecular Biotechnology Center “Guido Tarone”, University of Torino, via Nizza 52, 10126 Torino, Italy

**Keywords:** mantle cell lymphoma, relapsed/refractory, post-CART, monoclonal antibodies, bispecific antibodies

## Abstract

Mantle cell lymphoma (MCL) is a biological and clinical heterogeneous disease, ranging from more indolent subtypes to highly aggressive ones with a dismal prognosis. Bruton’s tyrosine kinase inhibitors (BTKis), such as ibrutinib, ameliorated the outcome of relapse/refractory (R/R) patients, but responses are usually quite short, requiring further treatments. In this setting, immunotherapy was demonstrated to have a key role, first with chimeric antigen receptor (CAR)-T cells, which completely changed the prognosis of these patients, showing a high rate of and long-lasting responses. However, many cases do not yet respond or relapse after CAR-T cells, and a subset of patients are not eligible for CAR-T cells in the first place. In this particular setting of MCL patients, next-generation therapies are emerging, such as new non-covalent BTKi molecules and novel monoclonal antibodies (MABs), which represent a heterogeneous group of agents, including Antibody-drug conjugate (ADC) and T-cell-engaging (TCE) products. The impact of these agents is expected to be meaningful firstly on relapse/refractory disease and subsequently also in early treatment lines. The aim of this review is to explore the main therapeutic strategies emerging in the context of MCL patients who failed or are not eligible for CAR-T-cells treatment, focusing on the most relevant therapeutic drugs, especially on the different monoclonal antibodies.

## Simple Summary

Mantle cell lymphoma (MCL) patients who were Relapsed/Refractory (R/R) after chimeric antigen receptor (CAR)-T cells or ineligible for that treatment had an unfavorable prognosis. This review aims to make an overview on the novel treatment emerging in this tough setting, such as new non-covalent BTKi molecules and novel monoclonal antibodies (MABs), including Antibody drugs conjugate (ADC) and T-cell-engaging (TCE) products.

## 1. Introduction

Mantle cell lymphoma (MCL) is a rare but heterogeneous subtype of non-Hodgkin lymphoma (NHL) with a prognosis ranging from very favorable in indolent cases to a poor prognosis for more aggressive variants [1]. Patients usually present with advanced-stage disease, with common extranodal involvement, including infiltration of bone marrow (53–82%), blood (50%), liver (25%), and the gastrointestinal tract (20–60%) [2,3].

Biologically, the pathognomonic signature is represented by cyclin D1 translocation and sometimes cyclin D2 or D3 translocations [4]. Clinical heterogeneity reflects partially a different cell of origin (COO): MCL can develop either from naïve-like cells and show a higher risk of progression or from memory-like cells of the adaptive immune system and present an indolent clinical course [5]. However, the most important regulator of proliferation in MCL is B-cell receptor (BCR) signaling [4,5]. According to different clinical behaviors and different COOs, the ICC/WHO 2022 update of lymphoid malignancies identified in MCL two distinct categories: nodal and non-nodal [4,6]. Nodal MCL (80–90% of cases) is characterized by unmutated immunoglobulin heavy-chain variable-region genes (IGHVs), Sex-Determining Region Y-Box 11 (SOX11) overexpression, COO by naïve-like cells, and generally more aggressive clinical behavior. Non-nodal leukemic MCL (10–20% of cases) typically displays mutated IGHV, SOX11 negativity, and COO from memory-like cells and presents with indolent biological behavior [4,6].

Histologically, three subtypes can be described: the “classical” MCL subtype and the pleomorphic and blastoid variants, with the variants displaying usually a more aggressive course and worse prognosis [3,4,6,7].

MCL has historically been considered an incurable disease, and the goal of management was often to prolong survival and reduce disease-related symptoms, in particular, in the relapsed/refractory (R/R) setting. In the last decades, the therapeutic landscape of MCL has been a topic of great interest, and molecular target validation has led to critical translational insights into novel therapies. Recently, results coming from large international trials have completely revolutionized both the frontline and R/R therapeutic approaches.

## 2. Prognostic Factors

Many clinical, serological, and biological factors are well known to be associated with worse prognosis in MCL. The MCL International Prognostic Index (MIPI) is based on four easy clinical factors—age, performance status, LDH, and leukocyte count—and demonstrated in many series high prognostic value [8]. In the MIPI score, each parameter has a different value, and it is able to stratify patients into low- (0–3 points, 44% of patients), intermediate- (4–5 points, 35% of cases), and high-risk (6–11 points, 21% of patients) groups, with statistically different median overall survivals: not reached vs. 51 months vs. 29 months, in the low- vs. intermediate- vs. high-risk group, respectively. There are other easy clinical features known to have prognostic value, such as advanced stage; splenomegaly; extranodal involvement; and the presence of B symptoms, anemia, or an elevated serum level of β2-microglobulin.

The MIPI score has been modified and implemented in different ways during the years. For example, the MIPI-c score has included the immunohistochemical determination of Ki-67 expression into the MIPI index, identifying in patients with a Ki-67 higher than 30% a worse prognostic group [9].

The proliferation rate, expressed as Ki-67 expression, and blastoid morphology have also proved reliable in the identification of a high-risk biology subgroup with significantly shorter survival and worse response to intensive chemotherapy regimens, with the particular relevance of the deletion of the short arm of chromosome 17 (del(17p)) or TP53 mutations, which have independent prognostic value in MCL patients [10]; this has been translated into the new lymphoma classification systems, whereby investigating the Ki67 proliferation index and TP53 status is mandatory at MCL diagnosis [6].

Furthermore, the minimal residual disease (MRD) status after treatment showed in many clinical trials a high prognostic value [11,12], but the application of MRD in clinical practice is still limited; also, the impact of MRD in the era of new targeted treatments, such as Bruton’s tyrosine kinase inhibitors (BTKis), remains a matter of open debate, particularly because BTKis are administered continuously until disease progression.

Recently, applying the whole-exome sequencing technique, four different biological clusters have been identified, with different outcomes.

Some additional data on new genomic analysis techniques, like optical genome mapping, are emerging, suggesting an impact of the burden of chromosomal aberration on patient prognosis, and NGS methodics are confirming the negative impact of TP53 mutations [13,14,15].

However, this stratification has not entered routine clinical practice yet.

## 3. New Milestones in MCL Treatment

Up to a few years ago, the standard of care in frontline treatment of MCL in young fit patients included an induction therapy with anthracycline and platinum-based chemotherapy regimens, followed by autologous stem cell transplantation (ASCT) consolidation for patients in complete remission (CR) [11,16]. In addition, a maintenance phase based on the anti-CD20 monoclonal antibody rituximab was demonstrated to improve survival rates if compared to observation alone [17]. As for older and/or unfit patients, backbone treatment with bendamustine-based chemoimmunotherapy followed by rituximab maintenance remains the standard of care [18,19]. An alternative chemoimmunotherapy regimen that could be considered in this setting is VR-CAP (Bortezomib, rituximab, cyclophosphamide, doxorubicin, and prednisone), which demonstrated high efficacy and a favorable safety profile in frontline MCL treatment [20] In relapsed MCL, monotherapies with BTKi, first of all Ibrutinib, have become the preferred salvage treatments in the second-line setting, based on their superior efficacy compared to conventional chemotherapy or other targeted agents [21,22,23]. Further than ibrutinib, other second-generation selective BTK inhibitors have been investigated alone and in combination in the setting of R/R MCL. First of all, Acalabrutinib is a second-generation, highly selective, and potent BTK inhibitor approved for the treatment of patients with R/R MCL who have received ≥1 prior therapies. It has been investigated as a single agent in R/R MCL in a large phase II trial (ACE-LY-004), demonstrating an ORR of 81.5%, with CR 47.6% [24,25]. Secondly, Zanubrutinib, which, like acalabrutinib, has been designed to overcome the limitation of the first-generation ibrutinib by delivering important and continuous inhibition of the BTK protein, thus optimizing bioavailability, half-life, and selectivity [26,27,28,29]. Zanubrutinib single agent in the setting of R/R MCL was investigated in multiple trials, showing high efficacy and a long duration of response, with a favorable safety profile [30]. In the phase II registry trial, Zanubrutinib single agent achieved an ORR of 83.7%, with 77.9% of CR and a median duration of response not reached, with median PFS of 33 months, in a cohort of R/R MCL patients [30] (Table 1).

On the basis of the high efficacy of BTKi, ibrutinib was investigated in frontline MCL treatment in addition to standard chemoimmunotherapy. In older patients, the addition of ibrutinib to bendamustine–rituximab (BR) was analyzed in the phase III double-blind randomized SHINE trial, where this combination was compared to BR plus placebo [31]. Among 523 patients, the addition of ibrutinib was demonstrated to significantly improve the CR rate and the progression-free survival (PFS) (80.6 months in the BR plus ibrutinib group and 52.9 months in the BR plus placebo group, HR 0.75, *p* = 0.01), though overall survival (OS) was similar in the two groups.

Multiple other attempts to ameliorate the standard frontline chemoimmunotherapy have been performed. One of the most relevant was the investigation of second-generation BTKi acalabrutinib in association with BR. The combination BR + acalabrutinib in previously untreated MCL was firstly studied in a phase Ib trial (ACE-LY-106) in both naïve and R/R MCL patients [32], where patients received acalabrutinib from cycle 1 until disease progression or treatment discontinuation; bendamustine on days 1 and 2 of each cycle for up to 6 cycles; and rituximab on day 1 of each cycle for 6 cycles, continuing every other cycle from cycle 8 for 12 additional doses in the first-line cohort. No new safety risks were identified, ORR was 94.4% and 85.0% in the frontline and R/R cohorts, respectively, while CR was 77.8% and 70.0%, respectively. At 47.6 months of follow-up, median PFS and OS were not reached in the treatment naïve group. Thus, a multicenter, double-blind, placebo-controlled phase III trial (ECHO trial) has followed, investigating this combination in a first-line setting, and has been recently presented at the last European Hematological Association (EHA) 2024 Meeting [33]. The study enrolled 598 patients (aged 65 or older), demonstrating a significant amelioration of PFS in the experimental arm (median PFS of 66.4 vs. 49.6 months, respectively, hazard ratio [HR] = 0.73; *p* = 0.0160) and a trend of better OS.

Another trial, which integrated new drugs in chemoimmunotherapy, is the FIL VR-BAC study [34]. In this trial, patients were allocated as low risk or high risk, depending on the tumor morphology (blastoid versus others), Ki67 expression (≥30% versus others), or presence of TP53 mutation and/or deletion. Patients with any of the three risk factors were classified as HR. Patients with low-risk disease were treated with six cycles of R-BAC (rituximab 375 mg/m^2^ d 1; bendamustine 70 mg/m^2^ d 1,2; cytarabine 500 mg/m^2^ d 1,2,3), while high-risk patients received abbreviated induction with 4 R-BAC, followed by consolidation (4 months, 800 mg/d) and maintenance (20 months, 400 mg/d) with venetoclax. This represents the first prospective study that stratified upfront patients with MCL to different treatments according to their risk profile. The results suggested that the addition of venetoclax to R-BAC improves the performance of the induction strategy, especially in high-risk patients, where the 2-year PFS and OS were, respectively, 58% and 66%, pointing to the importance of identifying high-risk patients from initial diagnosis.

However, all these regimens are based on the bendamustine backbone, that, even if it still represents the standard treatment for elderly MCL patients not eligible for high-dose therapy, opens important issues in the era of novel immunotherapies. In fact, bendamustine is well known to deteriorate T-cell compart and to damage the apheresis product for CAR-T manufacturing [35] A recent report clearly demonstrated that patients exposed to bendamustine had significantly lower CD3+ cells at apheresis and achieved lower ORR, shorter PFS and OS, if compared to not exposed patients. In bendamustine-exposed patients, those with recent treatment (within 9 months) had a worse prognosis. Based on this evidence, bendamustine-backbone treatment should be carefully evaluated in patients who could have CAR-T treatment in their near future, and a minimum time of 9 months from the last dose of bendamustine and apheresis would be desirable in order to not affect the prognosis [35].

In the setting of first-line treatment in young fit MCL patients, the international randomized phase III Triangle Trial has been recently published, representing a new milestone that modified the standard of care [36]. Patients with newly diagnosed stage II–IV MCL, aged 18–65 years and suitable for ASCT, were randomly assigned 1:1:1 to control group A (standard of care) or experimental groups A + I (standard of care with the addition of ibrutinib in both the induction and maintenance phases) or I (ibrutinib administered in the same way as in group A + I, but omitting ASCT). The 3-year failure-free survival (FFS) was significantly higher in the group A + I, if compared to the standard arm (88% vs. 72%, HR 0·52, *p* = 0·0008). The comparison of group A + I versus group I is ongoing to evaluate if there will still be a role for ASCT after the integration of ibrutinib in the first-line standard of care [26]. At the last ASH 2024, preliminary results of the comparison between arm A + I and I were shown, demonstrating no differences in FFS between the two arms. However, a trend towards the superiority of A + I over I has been suggested in very high-risk patients: Ki-67 > 30%, blastoid morphology, or high p53 expression [36]. On the other side, rituximab maintenance confirmed its benefit in terms of PFS in all the three arms [37].

Completely chemo-free regimens are emerging as another important new paradigm in treatment-naïve MCL patients. Three trials have recently been concluded. Firstly, the ENRICH trail, which investigated the combination of rituximab plus ibrutinib vs. chemoimmunotherapy regimens (R-bendamustine or R-CHOP) in previously untreated over-60-year-old MCL patients [38]. The results of this study were the first demonstrating an improved PFS for rituximab-ibrutinib (RI) versus immunochemotherapy in previously untreated MCL, and this goal was primarily driven by the improved PFS for RI versus RCHOP, while the PFS for IR versus BR was broadly similar. Subgroup analysis suggested a better performance of RI in the low-risk group (such as age < 70, ki67 < 30%, ECOG PS 0, non-blastoid variant, low MIPI score risk). Another important chemo-free experience is the BOVen trial, a phase 2 study of Zanubrutinib, Obinutuzumab, and venetoclax (BOVen) in untreated patients with MCL with a TP53 mutation. In the 25 patients included, the best ORR was 96% (24/25), and the CR was 88% (22/25), with 95% (18/19) of MRD negativity. The 2-year PFS and OS were 72% and 76%, respectively [39]. The third experience is the OAsIs trial (NCT02558816), as shown in Table 2, a single-arm multicenter prospective phase 1/2 trial that aimed to determine the maximum tolerated dose (MTD) of venetoclax in combination with fixed doses of ibrutinib and Obinutuzumab in relapsed MCL patients, with a cohort extension to untreated patients at the venetoclax MTD. Preliminary results showed a CR of 86.6% in untreated patients [40].

MCL is considered an incurable disease. Concerning the R/R MCL setting, huge efforts have been made in the last decade to address this urgent unmet clinical need. Development of Chimeric Antigen Receptor (CAR) T cells (CAR-T cells) completely changed the treatment landscape: in particular, the construct Brexucabtagene autoleucel (Brexu-cel) has been FDA, EMA, and AIFA approved as a third-line treatment for relapsed/refractory MCL, based on the results of the ZUMA-2 study [49,50], which showed that treatment with Brexu-cel induced a durable ORR of 91% and a median duration of response (DOR), PFS, and OS of 28.2 months, 25.8 months, and 46.6 months, respectively, in patients refractory or intolerant to BTKi treatment. These data were confirmed at a longer 3-year follow-up [50] and were demonstrated to be consistent with data coming from real-world practice (real-world evidence, RWE) [51]. Another CD19-directed CAR-T-cell product (Lisocabtagene maraleucel, Liso-cel) for R/R MCL has been evaluated in the phase 1 TRANSCEND NHL 001 study [52]. Results from this trial have recently been published, showing in 88 heavily pretreated patients with R/R MCL an ORR of 83.1%, with a CR rate of 72.3%, and a median PFS of 15.3 months, with a low incidence of grade ≥3 cytokine release syndrome (CRS), neurologic events (Immune effector cell-associated neurotoxicity syndrome, ICANS), and infections in a population of patients affected by high-risk, aggressive disease [51]. These favorable results have been confirmed in RWE, even if higher rates of adverse events emerged: CRS showed to be very frequent (>90% cases), but with a low incidence of grade >3 events; ICANS have been observed in 60–65% of cases, with 36% of grade >3 events [53]. Moreover, RWE showed a high rate of late hematological toxicities and infections risk. The non-relapse mortality rate was 10–15%, with infections as the main cause of death in these patients.

Also, an indirect comparison to a historical cohort of patients treated with chemoimmunotherapy showed a significant survival benefit with CAR-T therapy versus the standard of care in patients with R/R MCL after BTKi exposure [54], strongly suggesting that a CAR-T-cell approach should be offered to these patients.

Moreover, there is some evidence that immunomodulatory drugs, such as lenalidomide, could improve the depth and duration of the response to these T-cell engager treatments, and could be considered in this setting [55,56,57,58].

However, although a remarkable improvement in outcome has been achieved in recent years in both the frontline and R/RMCL, patients still experienced relapses, and a plateau in survival curves has not been reached yet. Here, next-generation therapies are emerging, such as new non-covalent BTKi molecules and novel monoclonal antibodies (MABs), which represent a heterogeneous group of agents, including Antibody–Drug Conjugate (ADC) and T-cell-engaging (TCE) products. The impact of these agents is expected to be meaningful, firstly on R/R disease and subsequently in early treatment lines too.

In this work we will analyze treatment options for MCL patients, who failed or are not eligible for CAR-T-cells treatment, with particular attention to drugs still under development.

## 4. Role of New Drugs in Relapsed/Refractory MCL After CAR-T Cells or Not Eligible for CAR-T-Cell Therapy

### 4.1. Currently Available Treatments

*BTK inhibitors*

*Pirtobrutinb*

The full mechanisms of resistance to BTKi are still unknown and controversial. The main mechanisms involved seem to be different in chronic lymphocytic leukemia (CLL) versus MCL [37]. In MCL, mutations of the cyclin D1 gene or the CDKN2A/MTAP gene could be the most important ones. Also, the onset of the acquired C481S mutation in the BTK enzyme, which prevents the formation of an irreversible covalent bond between the drug and enzyme, reducing the efficacy of BTKi, has been shown [59,60]. In this setting, Pirtobrutinib (LOXO-305), is a first-in-class, next-generation, highly selective, non-covalent, reversible BTKi with activity against C481-mutated BTK MCL and seems to be effective also in patients resistant to first-generation covalent BTKi.

Pirtobrutinib was investigated in a first-in-human pivotal multicenter phase 1/2 trial, which enrolled 13 patients (9 CLL and 4 MCL) with advanced disease (NCT03740529) (Table 3) [61]. Among patients evaluated for a response, pirtobrutinib treatment showed an 87.5% overall response rate, including a partial response (PR) in CLL patients with the C481 BTK mutation. No dose-limiting toxicities or grade 3 adverse events emerged [61].

Pirtobrutinib was then investigated in monotherapy in the setting of R/R MCL, in the BRUIN trial, an open-label, multicenter phase 1/2 study.

The trial included 61 patients with heavily pretreated R/R MCL. The median number of previous lines of therapy was three: 57 (93%) patients had received a previous BTKi, 60 (98%) an anti-CD20 antibody, 15 (25%) ASCT, 3 (5%) an allogeneic transplant, and 3 (5%) CAR-T-cell therapy. The treatment was demonstrated to be safe and manageable. The most common adverse events reported were fatigue, diarrhea, and contusion. Dose interruptions were observed in a minority of cases. Differently from covalent BTKi, with pirtobrutinib treatment, atrial arrhythmias and hemorrhage were rarely observed (two cases of atrial fibrillation, <1%, and only one case of reported grade 3 hemorrhage, a subarachnoid bleed sustained during a bicycle accident). After six months of follow-up, pirtobrutinib, at the dose of 200 mg daily, showed an ORR of 52%, with 25% of CR 25%, in patients with R/R MCL heavily pretreated and already exposed to BTKi. Responses were observed also in patients with a higher-risk variant (ORR 50% in blastoid variant cases) and in those who received previous cellular therapy, including nine (64%) patients with previous ASCT or allogeneic transplant, and 100% of cases with previous CAR-T-cell therapy. The response also was demonstrated to be early and sustained, with a median time to first response of 1.8 months, with a median duration of response of 8.3 months [41].

On this basis, in January 2023, the U.S. Food and Drug Administration (FDA) granted accelerated approval to pirtobrutinib for the treatment of adult patients with R/R MCL after at least two lines of systemic therapy, including a BTKi [62]. A randomized trial, BRUIN MCL-321 (NCT04662255) [63], is currently open to enrollment and evaluates pirtobrutinib vs. an investigator’s choice of BTKi (ibrutinib, acalabrutinib, or Zanubrutinib) in patients with MCL who have received at least one prior line of therapy and are BTKi-naïve. The primary endpoint is PFS, and secondary endpoints include OS.

*Ibrutinib + Venetoclax*
*Trial Sympatico*

Venetoclax is an oral BCL-2 inhibitor approved in the USA and Europe for the treatment of patients with CLL and previously untreated acute myeloid leukemia [64]. Venetoclax monotherapy was investigated in a phase 1 study in patients with R/R MCL, showing a 75% ORR and a 21% CR rate [42] (Table 2).

Ibrutinib and Venetoclax have distinct and complementary mechanisms of action and have demonstrated synergistic antitumor activity in preclinical models of MCL, showing significantly increase apoptosis (23%) in combinations when compared to each agent used alone (ibrutinib, 3.8%; venetoclax, 3.0%) [65].

On this encouraging basis, the phase 3 international study SYMPATICO (PCYC-1143-CA, NCT03112174) has been conducted, comprising an open-label Safety Run In (SRI) cohort and a subsequent double-blind randomized period, both including R/R MCL patients. An open-label arm in previously untreated patients is also currently ongoing [66,67] (Table 3).

The results of the primary analysis from the randomized, double-blind, phase 3 part of the SYMPATICO study comparing Ibr + Ven vs. Ibr + placebo (Pbo) in pts with R/R MCL were recently presented at the last 2024 American Society of Hematology (ASH) meeting. A total of 267 pts were enrolled and randomly assigned to receive Ibr + Ven (*n* = 134) or Ibr + Pbo (*n* = 133). The median age was 68 years old, with an ECOG PS of 0–1 in 96% of cases; 17% had at least three prior lines of treatment, and 22% were at increased risk for tumor lysis syndrome (TLS). With a median time of the study of 51.2 months, the median PFS was significantly longer with Ibr + Ven vs. Ibr + Pbo (31.9 vs. 22.1 months), with a hazard ratio (HR) of 0.65 (95% CI, 0.47–0.88; *p* = 0.0052). The 2y PFS was 57% and 45% with Ibr + Ven and Ibr + Pbo, respectively, with a consistent PFS benefit across high-risk subgroups, including those with a blastoid variant or *TP53*-mutated MCL. Grade ≥3 adverse events occurred in 84% of pts with Ibr + Ven vs. 76% with Ibr + Pbo, the most frequent being neutropenia (31% vs. 11%), pneumonia (13% vs. 11%), thrombocytopenia (13% vs. 8%), anemia (10% vs. 3%), diarrhea (8% vs. 2%), leukopenia (7% vs. 0%), and COVID-19 (5% vs. 1%). These data demonstrated that the Ibr + Ven combination led to a significant improvement in PFS compared with Ibr + Pbo in R/R MCL patients, and to a trend of improvement in OS, even if not statistically significant at this interim analysis. The safety profile of Ibr + Ven was consistent with known AEs for each agent, with no new safety signals observed, with an overall favorable benefit–risk profile for this combination in patients with R/R MCL [67,68].

### 4.2. Treatment Under Investigation but Not Currently Available

**Figure 1:**
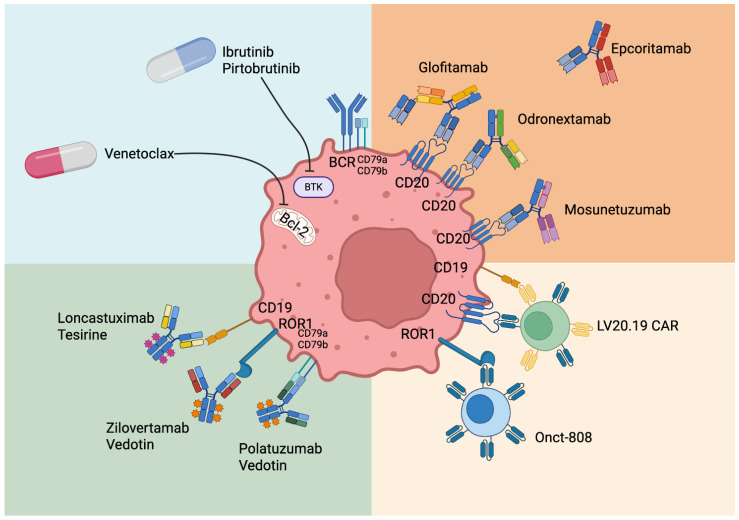
Main therapeutic targets and active drugs under investigation in mantle cell lymphoma.

A plethora of new targets are being evaluated for MCL (as summarized in Figure 1).

*Novel Antibodies*
*Antibody–drug conjugate*
*Zilovertamab Vedotin*

Zilovertamab vedotin is an immunoconjugate targeting ROR1, carrying the toxin monomethyl auristatin E [44,69,70] (Table 2).

In the first phase 1, first-in-human, dose-escalation study [70], 32 patients with previously treated lymphoid cancers were enrolled to receive Zilovertamab vedotin every 3 weeks until the occurrence of cancer progression or unacceptable toxicity had occurred. The 32 patients presented different histologies (MCL, CLL, diffuse large B-cell lymphoma (DLBCL), FL, Richter transformation lymphoma, or marginal zone lymphoma) and were heavily pretreated (median of four previous treatments). As expected with a monomethyl auristatin E-containing antibody–drug conjugate, adverse events included acute neutropenia and cumulative neuropathy, resulting in a recommended Zilovertamab vedotin dosing regimen of 2.5 mg/kg every 3 weeks. No clinically concerning adverse events occurred to suggest ROR1-mediated toxicities or non-specific binding to normal tissues.

Zilovertamab Vedotin induced objective tumor responses in 7 of 15 patients with MCL (47%; 4 partial and 3 complete) and in 3 of 5 patients with DLBCL (60%; 1 partial and 2 complete); objective tumor responses were not observed among patients with other tumor types.

At the last 2024 ASH, Glimelius et al. [44] presented the results of Cohort A of a phase II, open-label, multicohort, waveLINE-006 study (NCT05458297) of 40 relapsed/refractory MCL patients treated with Zilovertamab Vedotin. The median age was 68 years (range, 42–86), 28 pts (70%) were male, 19 (48%) had an ECOG PS of 0 or 1, 16 (40%) had a high MIPI risk, 7 (18%) were positive for the TP53 mutation, 22 (55%) had a KI67 proliferation fraction ≥30%, and the median number of prior lines of therapy was 4 (range, 2–9). All patients received a covalent or non-covalent BTKi. Eleven pts (28%) had undergone prior autologous stem cell transplant, and six (15%) received prior CAR-T-cell therapy. At the data cutoff, 37 pts (93%) had discontinued treatment (17 PD, 8 adverse even, 5 patient withdrawal, 4 clinical progression, 2 physician decision, 1 non-study anticancer therapy). The ORR was 40%: 5 pts (13%) had a CR and 11 (28%) achieved a PR. The median DOR was 3.0 months, with two responders with a response duration ≥ 6 months. The median PFS was 3.4 months, with a 6-month PFS of 26%. The median OS was 9.0 months, with a 6-month OS of 67%. Treatment-related AEs occurred in 36 pts (90%), of which the most common (≥25%) were neutropenia (58%), peripheral neuropathy (43%; due to conjugated monomethyl auristatine denned as peripheral neuropathy, paresthesia, peripheral sensory neuropathy, and polyneuropathy), and diarrhea (28%). Grade 3 or 4 treatment-related AEs occurred in 32 pts (80%), most commonly neutropenia and peripheral neuropathy. No pts died due to treatment-related AEs. These data confirmed that this setting of patients remains an unmet clinical need; however, the drug-conjugated antibody Zilovertamab vedotin showed promising results and a safe profile in the RR MCL setting, and further investigations are warranted.

*Loncastuximab-tesirine*

Loncastuximab-tesirine is another promising drug-conjugated monoclonal antibody, targeting CD19, conjugated with a pyrrolobenzodiazepine dimer [71,72]. This product was approved by the FDA in April 2021, and subsequently by EMA and AIFA, for the treatment of R/R large B-cell lymphoma (including DLBCL arising from low-grade lymphoma) in adult patients after two or more lines of immune chemotherapy, based on the LOTIS-2 trial [73]. In MCL, loncastuximab-tesirine, administered after a short course of chemotherapy, is under investigation in the COLUMN trial by Fondazione Italiana Linfomi (FIL), currently ongoing (NCT05249959) [74] (Table 2).

Another trial, investigating the safety and efficacy of the combination Loncastuximab Tesirine plus Ibrutinib in DLBCL and MCL (NCT03684694) [75], has recently concluded enrollment, and the results are expected in the near future.

Loncastuximab-tesirine has also been investigated in association with Durvalumab, a human monoclonal Ab of the immunoglobulin G-1 kappa subclass that blocks the interaction of programmed death-ligand 1 (PD-L1) with PD-1 on T cells and with CD80 (B7.1) on other immune cells, in a phase I/II trial (NCT03685344, [76]). Preclinical data, as well as early results from clinical trials combining ADC and checkpoint inhibitors, show the potentially increased effectiveness and synergism of these therapeutics when used in combination. The trial has recently concluded, and the results are expected soon.

Blockade of PD-L1/PD-1 and PD-L1/CD80 interactions releases the inhibition of immune responses, including those that may result in tumor elimination, and provides the rationale for the current trial.

Another ongoing trial is investigating the efficacy of Loncastuximab-tesirine in monotherapy in R/R B-cell malignancies, including MCL (NCT05453396) [48] (Table 2).

However, it is important to notice that Loncastuximab-tesirine treatment is burdened by important adverse events, which can limit its administration. Among them, we find gamma-glutamyltransferase increased (35.8%), neutropenia (34.9%), fatigue (30.2%), anemia (28.8%), thrombocytopenia (28.4%), nausea (26.5%), peripheral oedema (23.3%), and rash (20.0%) [48].

*Bispecific Antibodies*
*Glofitamab*

Glofitamab is a CD20 × CD3 bispecific antibody with an innovative 2:1 tumor–T-cell binding configuration, which translates into bivalent binding to CD20 on B cells and monovalent binding to CD3 on T cells, leading to T-cell engagement and redirection against malignant B cells [77]. Glofitamab is currently approved for the treatment of patients with R/R diffuse large B-cell lymphoma (DLBCL) after >2 lines of therapies [43,78].

Thus, Glofitamab was also investigated in the setting of MCL in the pilot phase I/II NP30179 study (NCT03075696) [79] (Table 2). In this trial, 61 MCL pts were enrolled and received Glofitamab with the standard ramp-up dose, premedicated with Obinutuzumab administered 7 days before the first glofitamab dose (single (1000 mg) or split over 2 days (2000 m) dose). Glofitamab step-up dosing was administered once a day on days 8 (2.5 mg) and 15 (10 mg) of cycle 1, with a target dose of 16 or 30 mg once every 3 weeks from cycle 2 day 1 onward, for 12 cycles. The 2000 mg dose of Obinutuzumab demonstrated reduction of the CRS rate and severity if compared to the single 1000 mg dose. Patients enrolled in the study were heavily pretreated, with a median of two previous therapies (range, 1–5): 51.7% had previously received a BTKi (58.1% of whom received it as their last previous therapy), 73.3% were refractory to their last line of previous therapy, and 36.7% had received previous treatment with bendamustine (22 patients, of whom 9 within the previous 12 months and 4 within the previous 6). With a median follow-up of 19.6 months, the treatment demonstrated high efficacy, with CR and ORR rates of 78.3% (65.8–87.9%) and 85.0% (73.4–92.9%), respectively. Responses were early and durable; the median time to the first response was 42 days, and the median duration of CR (DoCR) was 15.4 months (12.7-NE), with a 12-month DoCR rate of 71.0% (56.8–85.2). The median PFS and OS were 16.8 and 29.9 months, respectively. The patients who were BTKi-naïve showed significantly better outcomes if compared to BTK cases.

Likewise, responses were lower in patients who had received previous bendamustine. The CR and ORR rates were 68.2% (45.1–86.1) and 77.3% (54.6–92.2), respectively; however, these responses were still achieved even when bendamustine had been administered within the previous 12 months (7/9), even after CAR-T administration.

Toxicities were manageable and were most commonly CRS (70.0%), neutropenia (38.3%), COVID-19 (31.7%), and pyrexia (31.7%). Grade 3/4 AEs occurred in 65.0% of patients, most commonly neutropenia (23.3%), pneumonia (11.7%), anemia (11.7%), and CRS (11.7%); the CRS rate was lower in the cohort of patients receiving Obinutuzumab pretreatment 2000 mg vs. 1000 mg (63.6% vs. 87.5%), especially G > 3 events (6.8% vs. 25%). The median time to the CRS onset was 9.7 h and lasted for a median of 49 h after the first glofitamab dose (C1D8-C1D14) and 20.6 or 24.6 h after the second dose (C1D15-C1D21). CRS cases were well managed with the standard tocilizumab and corticosteroid therapy protocols and were especially needed in the Obinutuzumab 1000 mg group, as well as ICU admission (9.1% in the 1000 mg vs. 31.3% in the 2000 mg cohort).

ICANS presented in 11.7% (7/60) of patients and were all G1-G2 AEs and resolved easily.

Glofitamab was interrupted in 60% of patients due to AEs, mainly neutropenia (15%), CRS (10%), infections (COVID-19/COVID pneumonia) (15%), pneumonia 10%, and other infections (6.7%). Eight deaths were reported, all due to infections, though it is important to notice that six out of the eight deaths were COVID-19 related (COVID-19/COVID-19 pneumonia and one post-acute COVID-19 syndrome) due to the trial accrual during the pandemic.

Data on the Glofitamab efficacy in R/R MCL are encouraging; however, follow-up is still limited compared to, for example, with CAR-T, and more mature data are needed, above all on the duration of complete remissions; CD20 expression in this setting of heavily pretreated cases; and the best management of toxicities, such as CRS, though the impact of Obinutuzumab pretreatment on low levels of CRS should be weighted when comparing with other agents. Moreover, glofitamab efficacy needs to be evaluated in all subsets of patients, since a phase 1 pharmacodynamic analysis revealed that increased TP53 gene expression was associated with resistance to glofitamab, conveying a diminished effector T-cell profile [80].

Currently, the GLOBRYTE study is an ongoing phase III, open-label, multi-center, randomized, controlled trial evaluating the efficacy and safety of glofitamab monotherapy in patients with R/RMCL. This study compares glofitamab with the best investigator’s choice of rituximab + bendamustine (BR) or rituximab+ lenalidomide (R-Len) [81]. Results are expected in the near future.

*Epcoritamab*

Epcoritamab is another full-length IgG1 bispecific antibody redirecting CD3 + T cells to CD20-expressing cells [82,83,84]. Many data demonstrated Epcoritamab efficacy in monotherapy and in combinations in relapse/refractory DLBCL and follicular lymphoma. Epcoritamab is already approved in many countries for the treatment of R/R DLBCL after at least two previous lines of therapy. However, data on MCL are still limited: in the dose-escalation part of the pilot study in 73 patients with R/R NHL, only four MCL cases were included [85]. Thus, larger studies focusing on MCL histology are still needed.

*Mosunetuzumab single agent*

Mosunetuzumab is another humanized IgG1-based CD20 × CD3 BsAb with an altered Fc that does not bind to the complement or Fc gamma receptor. It has a single rituximab-like binding site for CD20 and a single binding site for CD3. Like in other bispecific antibodies, also the mosunetuzumab schedule included a dose-escalation phase to reduce the incidence and severity of CRS. The first application of Mosunetuzumab was treatment of R/R follicular lymphoma (FL), revealing a consistent efficacy. The pilot phase I–II study included patients with different subtypes of B-NHL, categorized into indolent non-Hodgkin lymphoma (iNHL); predominantly FL; and aggressive non-Hodgkin lymphoma (aNHL), predominantly DLBCL. Updated results of the study showed an ORR of 65.7 and 36.4%, with a CR rate of 49.3 and 21.7%, in iNHL and aNHL, respectively [86,87,88,89] (Table 3). The mPFS was 12.2 and 1.4 months for iNHL and aNHL, respectively. The efficacy is higher in the third- vs. later-line settings, while recent data demonstrated similar activity for both the disease progression within 24 months after frontline therapy (POD24) and not POD24. Based on early results from this study, mosunetuzumab received FDA and EMA approval in 2022 for the treatment of R/R FL after at least two prior lines of systemic therapy [90].

Mosunetuzumab single agent was also investigated in other settings, such as DLBCL [91,92], marginal zone lymphoma [93], or Richter Syndrome [94].

In MCL, mosunetuzumab monotherapy was studied in an expansion cohort in patients who are R/R to BTKi therapy, administered with the classical step-up dose [45] (Table 2). Twenty-five patients were enrolled, with a median age of 70 years (range: 50–89), Ann Arbor stage III/IV disease in 92%, and a MIPI score ≥6 in 84% of cases. The median number of prior therapies was 3 (range: 2–6), with one-third of cases having received previous ASCT, and 92% were refractory to their most recent regimen. At a median follow-up of 54.5 months, the best ORR and CR rates were 44% and 24%, respectively. Responses were demonstrated to be durable, with a median duration of response and of CR of 10.3 months and 18.0 months, respectively. The median PFS and OS were 3.7 and 7.3 months, respectively. The treatment showed a manageable safety profile, with the most common AEs CRS (52%, predominantly low grade and early), fatigue (36%), and pyrexia (36%). Suspected immune effector cell-associated neurotoxicity syndrome (ICANS) events occurred only in 3 pts (Grade 1 confusional state [*n* = 2]; Grade 2 delirium [*n* = 1]), two events concurrently with CRS. Grade 3/4 AEs were reported in 19 (76%) cases, including neutropenia (28%; no febrile neutropenia), hypophosphatemia (20%), thrombocytopenia (16%), and anemia (12%). Serious AEs (excluding fatal PD) were observed in fourteen (56%) pts; ten (40%) were treatment related. No grade 5 fatal AEs were reported. These data suggested mosunetuzumab single-agent activity in a challenging-to-treat population of pts with high-risk and refractory MCL. The safety profile was demonstrated to be manageable, and further investigations are ongoing.

*Mosunetuzumab combinations*

A Phase Ib/II study (NCT03671018), investigating Mosunetuzumab in combination with Polatuzumab-Vedotin (M-Pola) in patients with R/R B cell non-Hodgkin lymphoma, is ongoing (Table 3). Recently, the preliminary safety and efficacy analysis from the ongoing phase II expansion cohort of R/R MCL patients, who had received prior BTKi therapy, were presented [46,95]. Included patients had received ≥2 prior regimens (including an anti-CD20 agent, BTKi, and anthracycline- or bendamustine-based therapy). Mosunetuzumab was administered subcutaneously by step-up dosing on Days 1 (5 mg), 8 (45 mg), and 15 (45 mg) of Cycle 1, then 45 mg on D1 of every cycle from C2D1, every 3 weeks for a total of 17 cycles. Polatuzumab (1.8 mg/kg intravenous infusion) was administered on D1 of the first six courses. At this preliminary analysis, 20 patients had received M-Pola combination treatment. The median age was 68.0 (range 44–82) years, 95% had Ann Arbor stage III/IV disease, and 40% had a MIPI score ≥6 at study entry. The median number of prior lines of therapy was 3 (range 2–9). All cases had received prior BTKi therapy, 7 (35%) had received prior CAR-T-cell therapy, and 85% were refractory to their last therapy. Patients presented high-risk features, with 65% of cases with a Ki-67 proliferation index ≥50%, 50% with blastoid/pleomorphic variants, and 20% with demonstrated *TP53* mutation. The ORR and CR rates were 75% and 70%, respectively, with the median duration of CR not yet evaluable. The most common AEs were CRS (50%), fatigue (45%), dyspnea (35%), paresthesia (30%), diarrhea (30%), myalgia (30%), infusion-related reaction (25%), and nausea (25%). Two (10%) toxic deaths occurred, both due to COVID-19 pneumonia. Cases of neuropathy, all grade 1, occurred in three patients (15%). These data are encouraging about this treatment combination in heavily pretreated and very high-risk MCL patients.

*Odronextamab*

Odronextamab is another investigational off-the-shelf CD20 × CD3 bispecific antibody that demonstrated encouraging clinical activity in patients with heavily pretreated R/R diffuse large B-cell lymphoma (DLBCL) in the Phase 2, open-label, multicenter ELM-2 study (NCT03888105;) [96,97] (Table 2).

Thus, Odronexatamab was also investigated in the R/R MCL setting. The Phase 1 ELM-1 study (NCT02290951 [47]) was a single-arm, multicenter, phase 1, dose-escalation and dose-expansion trial, conducted at 10 academic sites across the USA and Germany. A total of 145 heavily pretreated NHL patients were enrolled in the trial, including 85 DLBCL, 40 follicular lymphoma (FL), 12 MCL, 6 marginal zone lymphoma, and 2 other NHL subtypes. In the cohort of 12 R/R MCL, Odronextamab demonstrated encouraging clinical activity, with an ORR of 50%. Patients with MCL showed a higher risk of cytokine release syndrome (CRS) when receiving T-cell engagers due to their circulating tumor burden and other high-risk features: this is reflected by the fact that in 12 patients receiving the initial 1/20 mg step-up regimen in ELM-1, 1 patient (1%) had a Grade 4 CRS event in the context of Grade 5 tumor lysis syndrome, and the global incidence of CRS was higher than in other histologies.

However, since its encouraging efficacy, also in a cohort of R/R MCL with a poor prognosis, that still remained an unmet clinical need; odronextamab is currently under investigation in this setting in the specific cohort of the phase II ELM-2 trial [96]. ELM-2 (NCT03888105) is an ongoing study of odronextamab in patients with R/R B-cell NHL that comprises five disease-specific cohorts, each with independent parallel enrollment: DLBCL, follicular lymphoma, MCL after BTKi therapy, marginal zone lymphoma, and other B-NHL. Patients are being recruited at sites across North America, Europe, and the Asia-Pacific regions. Eligibility criteria for the MCL cohort include age ≥18 years and R/R to ≥1 prior line of systemic therapy, including a BTKi. The MCL cohort will include 78 patients who will receive intravenous odronextamab monotherapy until disease progression or unacceptable toxicity. Following the high rates of observed Grade ≥3 CRS in MCL, including a fatal event in the ELM-1 trial, the step-up regimen was revised to 0.7/4/20 mg to help further mitigate the risk of CRS. At the last updated data presented, as of July 2024, 14 patients with MCL have been enrolled. Results from this trial are expected soon. A sub-analysis of this trial demonstrated that NHLs with greater programmed cell death-ligand 1 expression had a better response to odronextamab therapy; however, this work included only one patient with MCL, so the impact in this disease has not been established yet [98].

*Allogeneic stem cell transplantation*

Allogeneic stem cell transplantation (allo-SCT) can be considered as a post-CAR-T consolidation strategy in patients with relapsed/refractory (R/R) MCL, although prospective evidence is limited [99]. Previous retrospective studies have suggested that allo-SCT may offer a graft-versus-lymphoma (GVL) effect, potentially leading to more durable remissions. In a Spanish retrospective series of 135 patients undergoing allo-SCT, a 5-year event-free survival (EFS) of 47% and an OS of 50% were reported [100]. In this study, the incidence of chronic graft-versus-host disease (cGVHD) was significantly higher in patients who achieved a CR post-transplant (43%) compared to those who did not (0%). The results also demonstrated that chemo-refractoriness is not a significant risk factor for disease control after allo-SCT. The relapse rate was low (7% and 12% at 1 and 3 years, respectively), but the high non-relapse mortality (NRM) may have reduced the number of patients at risk of relapse, since the cumulative incidence of day 100 and one-year NRM were 17% and 32%, respectively. The median age of the patients was 55 years, and the prior lines of therapy were 1–2 in 55% of cases and >2 in 37% of cases. Conditioning was myeloablative in 48% of cases and reduced-intensity conditioning (RIC) in 47% of cases. This series, however, did not include patients with prior exposure to CAR-T-cell therapy; moreover, patients relapsed after CAR-T may be older, with a possible impact on the conditioning regimen, toxicity, and survival.

Di Blasi et al. [101] recently described 238 patients with relapsed/refractory aggressive B-cell lymphomas after CAR-T (both axi-cel and tisa-cel) in France. Relapse/progression was classified as very early (before d +30 days after CAR-T), early (between d +31 and d + 90), and late (>d + 90). The ORR and CR were 14% and 65%, respectively, with the median survival range from 3.7 to 8.5 months. The LDH, ferritin, and CRP levels at infusion and very early relapse were predictive factors for outcomes. To note, no association between previous treatment types and outcomes was observed. However, this cohort lacked MCL patients.

Furthermore, allo-SCT is still complicated by a significant NRM due to infections and GVHD, and can be complicated by the aggressiveness of the disease, poor patient performance status, and/or cytopenias, which can preclude the administration of induction therapy [101]. Liebers et al. [102] compared patients treated with brexucel in the ZUMA-2 study with EBMT MCL patients who underwent allo-SCT by propensity score: at the almost 3-year follow-up, brexucel patients had a significantly higher 1-year OS (81.3% vs. 59.2%, *p* = 0.004) and lower NRM (3.6% vs. 21.2%, *p* = 0.015), with cGVHD in 26.9% of alloHCT patients within the first year. However, the long-term progression-free survival and OS remain comparable.

In another work on B-NHL, 16 patients were transplanted after CAR-T failure, with a median PFS and OS of 14.3 and 16.2 months, respectively, from the time of transplant, and the estimated 1-year PFS and OS were 52% and 66%, respectively, with promising results in patients who had achieved a CR at the time of allo-SCT at the 24-month follow-up (6 out of 7 CR still ongoing). However, none of these patients were MCL (13 de novo DLBCL, 2 transformed indolent lymphomas, 6 PMBCL) [103].

Moreover, another experience on allo-SCT in DLBCL reported comparable efficacy, with a 2-year PFS and OS of 31% and 45%, respectively, at the 32-month follow-up but at the cost of considerable toxicity, especially hepatic (28%) and sinusoidal obstruction syndrome (15.4%), and a 2-year non-relapse mortality and relapse/progression incidence of 26% and 43%, respectively [104].

Another encouraging result was reported by Iacoboni et al., where the median OS for 32 patients with R/R DLBCL transplanted after CAR-T was not reached at the 15.1-month follow-up, with a 12-month OS of 84% [105].

Thus, the decision to proceed with allo-SCT after CAR-T-cell therapy must be carefully evaluated, considering the patient’s fitness, the availability of a suitable donor, and the risk of NRM. Further research is needed to establish the optimal criteria for patient selection and the timing of allo-SCT after CAR-T-cell therapy in R/R MCL.

*Horizon Scanning and Future Perspectives*
*BTK degraders*

The continuous treatment with BTKi put selective pressure, leading to multiple BTK mutants. In this setting, a new class of BTK-targeting drugs emerge: the BTK protein degraders, which use the ubiquitin–proteasome pathway to ubiquitinate BTK protein in the cell, leading to proteasomal destruction of BTK. Preclinical experiments show broad activity of BTK protein degraders across the spectrum of wild-type and mutant BTKs, including the most frequent ones identified as mechanisms of resistance in CLL and MCL [106].

The primary results came from the first-in-human drugs NX-2127 and NX-5948.

NX-2127 was investigated in a phase I/Ib trial, with a dose escalation phase and an expansion cohort, enrolling relapsed/refractory B-cell malignancies, including CLL (40 cases, dose 100 mg), MCL (20 patients, dose 300 mg), and DLBCL (20 patients, dose 300 mg) [107] (Table 2).

NX5948 is under investigation in a phase I/Ib trial including CLL, MCL, marginal zone lymphoma, Waldenstrom Disease, follicular lymphoma, DLBCL, and Primary Central Nervous System Lymphoma (Table 2) [108].

Preliminary results of these studies suggested that BTK degraders show early efficacy in clinical trials, with a manageable safety profile that was consistent with previous reports for BTK-targeted therapies, overcoming common resistance mechanisms to kinase inhibitors.

*Novel Tyrosine Kinase (TK) inhibitors*

Narazaciclib (ON123300) is a second-generation, orally bioavailable, and clinical-stage CDK4/6 inhibitor (CDKi) that may trigger cell cycle arrest and significant tumor growth inhibition (TGI) in BTKi-resistant MCL [109]. It was demonstrated to be safe and effective in in vitro and in vivo models of MCL [110].

KIN-8194, a Src-family tyrosine kinase hematopoietic cell kinase (HCK) inhibitor, arrests growth and the integrin-mediated adhesion of BTKi-sensitive MCL cells, as well as MCL cells with primary or acquired BTKi resistance, and faces the therapeutic landscape as a promising novel treatment for MCL patients.

*Novel BCL-2 inhibitors*

Several preclinical studies have suggested BCL-2 as a possible co-target for R/R MCL, and the SYMPATICO study has paved the way for the testing of new BCL-2 inhibitors, often to overcome venetoclax resistance [111,112,113]. The efficacy of obataclax, a BH3 mimetic inhibitor of anti-apoptotic Bcl-2 proteins, which demonstrated synergy with bortezomib in preclinical models, was studied in a phase 1–2 trial in association with bortezomib for 13 R/R MCL patients. The combination was overall well tolerated; however, the ORR was 31% (4/13), and the increased effectiveness compared to bortezomib alone was not confirmed [114].

Another molecule investigated is BGB-11417 (sonrotoclax), a highly selective Bcl-2 inhibitor with 5-fold increased potency in pharmacodynamic studies [115]. Phase I data in patients with R/R MCL showed an ORR of 55% when used in combination with Zanubrutinib (Table 2) [116].

Further studies are needed to confirm these promising results in a larger population.

*AKT-inhibitors*

Capivasertib is a novel oral pan-AKT inhibitor, which has been studied in a phase II trial including 2 MCL patients among the 15 total patients. The authors reported an ORR of 54% and a CR rate of 8%. A grade 3 AE occurred in three patients (20%), without a G ≥ 3 infection, displaying a manageable safety profile in heavily pretreated NHL [117].

*Novel CAR-T cells*

Another promising field of research is investigating novel CAR-T-cells constructs, direct towards new targets.

In this landscape, of particular interest is a new anti-ROR1-specific CAR-T cell (Onct-808), which has been investigated in a phase 1/2 multicenter study in patients with R/R aggressive B-cell lymphoma [118]. At the last European Hematology Association (EHA) meeting, the results on the first four enrolled patients were presented. Of the four treated patients, three cases had R/R MCL, and two patients received prior CD19 CAR-T cells. Toxicities were relevant, with one 80-year-old patient dying from CRS and ICANS complications, and two cases of grade 3 pneumonia, and due to clinical and economic reasons, the trial was terminated. Response assessments were evaluated in three pts, with CR observed at Month 1 for two of three pts, while the third experienced PR.

Another promising product investigated in MCL is LV20.19 CAR, a bispecific, tandem, lentiviral CAR-T cell targeting both CD20 and CD19 B-cell antigens, with 4-1BB co-stimulatory signaling. This peculiar construct will improve an expansion in IL7 and IL15, producing less “exhausted” CAR product, resulting in more durable clinical activity. Shah et al. conducted a phase 1/2 single-center, prospective trial (NCT04186520) evaluating LV20.19 CAR-T cells at a fixed dose of 2.5 × 10^6^ cells/kg for patients with R/R NHL and presented the results of the MCL cohort [119]. Ten MCL patients received LV20.19 CAR-T cells; manufacturing was successful in 100% of cases, and all received fresh (non-cryopreserved) product. The clinical characteristics were median age of 62 years, with a median of 4 (range 3–8) previous lines of therapy. All patients were heavily pretreated: three cases received prior ASCT and two patients allogeneic HCT; 80% had progressed on a BTKi, and four of these patients had additionally progressed on a non-covalent BTKi (pirtobrutinib) administered on a clinical trial. The Day 28 ORR was 100% (CR = 60% and PR = 40%). Seven of nine pts with MRD assessed between Day 28 and 60 post-CAR were negative. No patient has relapsed, with a median follow-up of 18 months among surviving pts. Regarding the toxicity profile, 100% of patients experienced a Grade 1–2 CRS (but no grade >3 events were observed). ICANS occurred in two cases, with one patient experiencing Grade 2 and the other Grade 3 toxicity. At 1 year, the cumulative incidence of relapse and NRM were 0% and 10% (one patient died because of gram-negative sepsis after the Day 28 evaluation), respectively, while the 1-year PFS and OS were 90% each. These data suggested that dual targeting of CD19 and CD20 with CAR-T cells may improve outcomes in R/R MCL patients; but data on a larger cohort and longer follow-up are needed [120].

Many other products are on the horizon, scanning for R/R MCL, such as Milatuzumab, a fully humanized anti-CD74 monoclonal antibody, which demonstrated significant activity in preclinical lymphoma models but failed to provide meaningful benefits in clinical trials, mainly due to its short half-life. This could be overcome targeting CD74 using a CAR-T cell; that would probably provide potent and durable anti-MCL activity [121]. Further studies in this direction are ongoing.

*Exploratory trials*

Several new targets are being investigated in a number of active phase I–II trials, both as single agents and in combination with other drugs, both already in use and experimental, as shown in Table 2.

They include, in particular, copanlisib (PI3K inhibitor, NCT04939272), linperlisib (PI3Kδ inhibitor, NCT06324994), alisertib (selective aurora A kinase inhibitor, NCT01695941), avelumab (anti PD-L1 MAB) and utomilumab (anti-human 4-1BB MAB, NCT03440567), nemtabrutinib (novel BTKi, NCT03162536), rocbrutinib (novel BTKi, NCT05716087 and NCT04775745), CYT-0851 (MCT-mediated lactate transport inhibitor, NCT03997968), NX-2127 (BTK degrader, NCT04830137) and NX5948 (BTK degrader, NCT05131022), TQB3909 (BCL-2 inhibitor, NCT06106841), novel anti-CD19 CAR-T (NCT04484012), anti-ROR1 CAR-T (NCT05444322), CLIC-2201 (anti-CD22 CAR-T, NCT06208735), NVG-111 (ROR1-CD3 bispecific antibody, NCT04763083), anti-BAFFR CAR-T (NCT05370430), CD19 CAR NK/T (NCT06464861), SynKIR-310 (anti-CD19 KIR CAR-T, NCT06544265), SC262 (anti-CD22 CAR-T, NCT06285422), GLPG5101 (anti-CD19 CAR-T, NCT06561425), ATA3219 (allogeneic anti-CD19 CAR-T, NCT06256484), CAR.k.28 (NCT04223765), iopofosine (phospholipid drug conjugate delivering I-131, NCT02952508), pemigatinib (FGFR2 inhibitor, NCT06300528), CTX112 (allogeneic CRISPR-Cas9 engineered anti-CD19 CAR-T, NCT05643742), voruciclib (CDK9 inhibitor, NCT03547115), AC676 (BTK degrader, NCT05780034), AZD5492 (CD8-Guided T-Cell Engager, NCT06542250), and PRT2527 (CDK9 inhibitor, NCT05665530); and already mentioned drugs like pirtobrutinib, in addition to glofitamab (NCT06252675), BGB-11417 (novel BCL-2 inhibitor, NCT05471843), LV20.19 CAR-T (NCT04186520), loncastuximab (anti-CD19 ICD, NCT05249959 and NCT05453396), and odronextamab (NCT03888105).

Some trials also use already known drugs, such as carfilzomib and ixazomib (proteasome inhibitors), lenalidomide (immunomodulatory drugs), polatuzumab vedotin (anti-CD79b ICD), tafasitamab (anti-CD19 MAB), nivolumab (anti-PD-1 MAB), oral azacitidine (demethylating agent), anti-CD20 CAR-T (NCT03277729), blinatumomab (anti-CD19 MAB), and Zanubrutinib (BTKi). The first results of these pioneering trials are eagerly awaited.

## 5. Conclusions

In recent years, remarkable progress in the immunotherapy of hematologic malignancies has significantly improved outcomes for patients with R/R lymphoid diseases. In mantle cell lymphoma, the anticipation of BTK inhibitors in the first-line setting and the spreading of CAR-T-cells therapy completely changed the landscape. However, many cases do not yet respond or relapse after CAR-T cells, and a subset of patients are not eligible for CAR-T in the first place. This setting of MCL patients still represents a challenge and an unmet clinical need. Next-generation therapies are emerging, such as new non-covalent BTKi molecules and novel monoclonal antibodies (MABs), which represent a heterogeneous group of agents, including Antibody drug-conjugated (ADC) and T-cell-engaging (TCE) products. The impact of these agents is expected to be meaningful, firstly on relapsed/refractory (R/R) disease and subsequently also in earlier treatment lines.

## 6. Future Directions

The near future in mantle cell lymphoma will see the employment of BTK inhibitors in frontline treatment. Also, biological high-risk features, such as TP53 mutations or the ki67 proliferation index, will help, maybe more than age and fitness, to guide clinicians in the treatment choice. The landscape of R/R patients who failed or were ineligible for CAR-T cells still remains a challenge, and novel monoclonal antibodies and novel combinations will probably play a key role in this setting.

**Figure 2:**
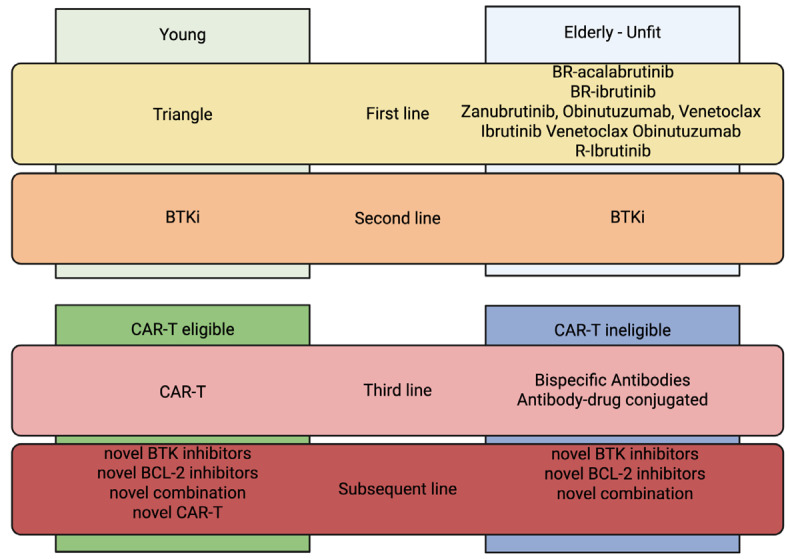
Proposed therapeutic algorithm for mantle cell lymphoma including both clinically available and experimental drugs, expected in the near future. Abbreviations—BR: bendamustine rituximab; BCL: B-cell lymphoma; BTKi: Bruton tyrosine kinase inhibitor; CAR-T: chimeric antigen receptor-T cell; R: rituximab.

In this scenario (Figure 2), future treatment sequencing for young and/or fit patients may be considered as chemoimmunotherapy plus ibrutinib frontline; at the first relapse, CAR-T cells are not allowed in the present moment but may be anticipated in the close future. Actually, in the second line, we can consider BTKi again; eventually non-covalent BTKi, such as pirtobrutinib; followed by CAR-T cells at the second relapse. In elderly patients, first-line treatment is still mainly represented by chemoimmunotherapy with bendamustine-containing regimens, with eventual addition of BTKi (ibrutinib or acalabrutinib), which may impact the later efficacy of TCE, especially in early relapse at less than 9 months from the last bendamustine dose. In the near future, we may have chemo-free regimens available. The second-line therapy can be still represented by BTKi. In patients who fail CAR-T cells, or who are not eligible for that kind of intensive treatment, bispecific antibodies, even if at the present moment not available yet, will probably achieve a key role in the future, especially in early CD20-positive relapses, followed by an ADC like zilovertamab-vedotin or loncastuximab as a last resort. Novel therapies, such as BTK-degraders or novel BCL2 inhibitors and novel combinations, still need to be deeper investigated but will probably have a growing role in the close future in R/R MCL patients, moreover, with progressive anticipation of other drugs in earlier therapeutic lines.

## Figures and Tables

**Table 1:** Comparison of covalent Bruton Tyrosine Kinase inhibitors in mantle cell lymphoma.

Drug	Study	Patient Number	Post-ASCT	Post-CAR-T	ORR	CR	OS	mPFS	mDOR	mFU	AEs ≥ G3	Ref
Ibrutinib	NCT01236391	111	12 (11%)	-	68%	21%	N.R.	13.9	17.5	15.3		[22]
Ibrutinib	NCT01646021	280	-	-	72%	19%	N.R.	14.6	N.R.	20	94 (68%)	[23]
Acalabrutinib	NCT02213926	124	22 (18%)	-	81%	40%	N.R.	N.R.	N.R.	15.2	48 (39%)	[24]
Zanubrutinib	NCT03206970	86	-	-	83.7%	77.9%	N.R.	33	N.R.	35.3		[30]

Abbreviations—AEs ≥ G3: adverse events of grade equal or above 3; ASCT: autologous stem cell transplant; CAR-T: chimeric antigen receptor-T cell; CR: complete response; mDOR: median duration of response; mFU: median follow-up; mPFS: median progression-free survival; NR: not reached; ORR: overall response rate; OS: overall survival.

**Table 2:** Available data on potential drugs in patients with R/R MCL after CAR-T failure or in patients ineligible for CAR-T.

	Study	Patient Number	Post-ASCT	Post-CAR-T	ORR	CR	OS	mPFS	mDOR	mFU	AEs ≥ G3	Ref
Pirtobrutinib	NCT03740529	127			81%	43%	NR 62% at 18 months	NR 74% at 18 months		18	(20.5%)	[41]
Venetoclax + ibrutinib	NCT03112174	267	39 (29%)		75%	21%	36.7	31.9			(84%)	[42]
Glofitamab	NCT03075696	61	2 (3.3%)	16 (26.7%)	74%	71%	29.9	16.8	16.2	19.6	(65%)	[43]
Zilovertamab vedotin	NCT03833180	40	11 (28%)	6 (15%)	40%	13%	9	3.4	3		32 (80%)	[44]
Mosunetuzumab	NCT02500407	25	(33%)		44%	24%	7.3	3.7	10.3	54.5	19 (76%)	[45]
Mosunetuzumab + polatuzumab	NCT03671018	20	15 (12.5%)	42 (35%)	75%	70%	23.3	11.4	NR at mFU of 45 months	45	(56.7%)	[46]
Odronextamab	NCT02290951	12		3 (25%)	50%	33%			7.6	26.2		[47]
Loncastuximab-tesirine	NCT02669017	15			46.7%	33%	N.R.	4.8				[48]

Abbreviations—AEs ≥ G3: adverse events of grade equal or above 3; ASCT: autologous stem cell transplant; CAR-T: chimeric antigen receptor-T cell; CR: complete response; mDOR: median duration of response; mFU: median follow-up; mPFS: median progression-free survival; NR: not reached; ORR: overall response rate; OS: overall survival.

**Table 3:** Ongoing trials after BTKi and CAR-T in R/R MCL.

NCT Number	Name	Phase	State	Drug
NCT04939272		I–II	Not recruiting	Copanlisib Hydrochloride, Venetoclax
NCT03891355	FIL_KLIMT	II	Not recruiting	Carfilzomib, Lenalidomide, Dexamethasone
NCT04047797		II	Not recruiting	Ixazomib, Ixazomib Citrate, Rituximab
NCT02558816	OAsls	I–II	Not recruiting	Ibrutinib, Obinutuzumab, GDC-0199 (Venetoclax)
NCT02446236		Ib	Not recruiting	Lenalidomide, Ibrutinib, Rituximab
NCT01695941		I	Not recruiting	Alisertib, Bortezomib, Rituximab
NCT03440567		I	Not recruiting	Autologous Hematopoietic Stem Cell Transplantation, Avelumab, Carboplatin, Etoposide Phosphate, Ibrutinib, Ifosfamide, Rituximab, Utomilumab
NCT04659044		II	Not recruiting	Polatuzumab Vedotin, Rituximab, Rituximab and Hyaluronidase Human, Venetoclax
NCT01996865	MAGNIFY	IIIb	Not recruiting	Lenalidomide, Rituximab
NCT04703686		II	Not recruiting	Obinutuzumab, RO7082859 (Glofitamab)
NCT03162536		I–II	Not recruiting	Nemtabrutinib
NCT03015896		I–II	Not recruiting	Lenalidomide, Nivolumab
NCT04578600		I/Ib	Not recruiting	Lenalidomide, Obinutuzumab, Oral Azacitidine
NCT03277729		I/II	Not recruiting	Chimeric Antigen Receptor-T-Cell Therapy, Cyclophosphamide, Fludarabine Phosphate
NCT04205409		II	Not recruiting	Nivolumab
NCT02568553		I	Not recruiting	Blinatumomab, Lenalidomide
NCT03997968		I/II	Not recruiting	CYT-0851, CYT-0851 + gemcitabine/capecitabine/rituximab and bendamustine
NCT05131022		Ia/Ib	Recruiting	NX5948
NCT06252675		II	Recruiting	Obinutuzumab, Glofitamab, Pirtobrutinib
NCT05471843		I/II	Not recruiting	BGB-11417
NCT05868395		II	Recruiting	Polatuzumab, Bendamustine, Rituximab
NCT06324994		II	Not yet recruiting	Linperlisib + Obinutuzumab and Venetoclax
NCT06106841		Ib/II	Recruiting	TQB3909
NCT04484012		II	Recruiting	Acalabrutinib, CD19CAR-CD28-CD3zeta-EGFRt-expressing Tn/mem-enriched T lymphocytes
NCT04186520		I/II	Recruiting	LV20.19 CAR-T
NCT05910801		II	Recruiting	Lenalidomide, Tafasitamab, Venetoclax
NCT05716087		II	Not recruiting	LP-168 (Rocbrutinib)
NCT06192888		I	Recruiting	Glofitamab, Obinutuzumab, Lenalidomide
NCT06558604	GLOASIS	II	Recruiting	Obinutuzumab, Glofitamab, Venetoclax, Zanubrutinib
NCT05249959	COLUMN	II	Recruiting	ADCT-402 (loncastuximab tesirine), Rituximab-Bendamustine, Ara-C
NCT05529069		II	Recruiting	Pirtobrutinib, Venetoclax
NCT05444322		I	Recruiting	RD14-01 cell infusion (anti ROR1 CAR-T)
NCT06300528		II	Recruiting	Pemigatinib
NCT05990465		I	Recruiting	Pirtobrutinib, LV20.19 CAR-T
NCT06208735		I	Recruiting	CLIC-2201
NCT04763083		I	Recruiting	NVG-111
NCT05370430		I	Recruiting	BAFFR-CAR-T cells
NCT06464861		I	Recruiting	CD19-CAR-NK/T
NCT06544265		I	Recruiting	SynKIR-310 (Autologous T Cells Transduced with CD19 KIR-CAR)
NCT03676504		I/II	Recruiting	CD19.CAR-T Cells, Fludarabine, Cyclophosphamide
NCT04775745		I	Recruiting	LP-168 (Rocbrutinib)
NCT05643742		I/II	Recruiting	CTX112 (Allogeneic CRISPR-Cas9 Engineered CD19 CAR-T)
NCT05453396		II	Recruiting	Loncastuximab Tesirine
NCT06285422		I	Recruiting	SC262
NCT05887167		I	Recruiting	Autologous Stem Cell Transplantation, CAR-T
NCT04830137		Ia/Ib	Recruiting	NX-2127
NCT02952508	CLOVER-1	II	Not recruiting	CLR 131 (Iopofosine I 131)
NCT06191887		Ia/Ib	Recruiting	Autologous BAFFR-targeting CAR-T Cells, Bendamustine, Cyclophosphamide, Fludarabine
NCT06561425		I/II	Recruiting	GLPG5101
NCT03888105		II	Recruiting	Odronextamab
NCT03547115		I	Recruiting	Voruciclib
NCT06256484		I	Recruiting	ATA3219
NCT04223765		I	Recruiting	CAR.k.28, Fludarabine, Cyclophosphamide, Bendamustine
NCT05780034		I	Recruiting	AC676
NCT06542250	TITANium	I/II	Recruiting	AZD5492
NCT05665530		I	Not recruiting	PRT2527, Zanubrutinib, Venetoclax

## Data Availability

No new data were created or analyzed in this study. Data sharing is not applicable to this article. The original contributions presented in this study are included in the article. Further inquiries can be directed to the corresponding author(s). Images created in BioRender. Comba, L (2025). https://biorender.com/edv7htl (accessed on 26 June 2025); https://biorender.com/yv371pa (accessed on 26 June 2025).

## Abbreviations

MCL	mantle cell lymphoma
CAR-T cells	chimeric antigen receptor-T cells
BTKi	Bruton’s tyrosine kinase inhibitor
MAB	monoclonal antibody
ICD	immunoconjugated drug
TCE	T-cell engaging
R/R	relapsed/refractory
NHL	non-Hodgkin lymphoma
COO	cell of origin
MIPI	MCL international prognostic index
MRD	minimal residual disease
ASCT	autologous stem cell transplantation
ORR	overall response rate
CR	complete remission
PFS	progression-free survival
OS	overall survival
DOR	duration of response
BR	Bendamustine-rituximab
BAC	Bendamustine-cytarabine-rituximab
R-CHOP	rituximab-prednisone-doxorubicin-vincristine-cyclophosphamide
MTD	maximum tolerated dose
Brexu-cel	Brexucabtagene autoleucel
Liso-cel	Lisocabtagene maraleucel
CLL	chronic lymphocytic leukemia
TLS	tumor lysis syndrome
CRS	cytokine release syndrome
ICANS	immune effector cell-associated neurotoxicity syndrome
ROR1	retinoic acid receptor 1
PD-L1	programmed death-ligand 1
DLBCL	diffuse large B-cell lymphoma
FL	follicular lymphoma
